# A novel autoantibody against ephrin type B receptor 2 in acute necrotizing encephalopathy

**DOI:** 10.1186/1742-2094-10-128

**Published:** 2013-10-18

**Authors:** Tsuyoshi Shirai, Hiroshi Fujii, Masao Ono, Ryu Watanabe, Yuko Shirota, Shinichiro Saito, Tomonori Ishii, Masato Nose, Hideo Harigae

**Affiliations:** 1 Department of Hematology and Rheumatology, Tohoku University Graduate School of Medicine, 1-1 Seiryo-cho, Aoba-ku, Sendai, Miyagi 980-8574, Japan; 2 Department of Histopathology, Tohoku University Graduate School of Medicine, 1-1 Seiryo-cho, Aoba-ku, Sendai, Miyagi 980-8574, Japan

**Keywords:** Acute necrotizing encephalopathy, Autoantibody, Ephrin type B receptor 2, Serological identification system for Autoantigens using a Retroviral vector and Flow cytometry (SARF)

## Abstract

Acute necrotizing encephalopathy (ANE) is characterized by symmetrical brain necrosis, suggested to be due to breakdown of the blood–brain barrier (BBB). We experienced a rare case of ANE complicated with systemic lupus erythematosus (SLE), and found that the patient’s serum (V10-5) had binding activity to human umbilical vein endothelial cells (HUVECs). By SARF (Serological identification system for Autoantigens using a Retroviral vector and Flow cytometry) method using V10-5 IgG, a clone bound to V10-5 IgG was isolated. This cell clone was integrated with cDNA identical to *EphB2*, which plays critical roles in neuronal cells and endothelial cells. HUVECs and human brain microvascular endothelial cells expressed EphB2 and the V10-5 IgG bound specifically to *EphB2*-transfected cells. Anti-EphB2 antibody was not detected in other SLE patients without ANE. In this report, we identified EphB2 as a novel autoantigen, and anti-EphB2 antibody may define a novel group of brain disorders. Anti-EphB2 antibody can interfere not only with endothelial cells including those of the BBB (acting as an anti-endothelial cell antibody), but also neuronal cells (acting as an anti-neuronal antibody) if the BBB has been breached. Future studies should determine the clinical prevalence and specificity of anti-EphB2 antibody, and the molecular mechanisms by which anti-EphB2 antibody mediates neuronal and vascular pathological lesions.

## Background

A group of brain disorders involving autoantibodies targeting cell-surface or synaptic proteins has been identified. The antigens include N-methyl-D-aspartate receptor (NMDAR), α-amino-3-hydroxy-5-methyl-4-isoxazolepropionic acid receptor (AMPAR), γ-aminobutyric acid receptor-B (GABAB receptor), leucine-rich glioma-inactivated protein 1 (LGI1), contactin-associated protein-like 2 (Caspr2), glycine receptor (GlyR), and metabotropic glutamate receptor mGluR5 [1].

Autoantibodies cause tissue damage through a number of mechanisms. Especially, cell-surface target antigens are susceptible to disruption by autoantibodies, and syndromes mediated by these autoantibodies often mimic animal models with genetic or pharmacological disruption of these molecules [1]. Identifying these autoantigens would be helpful in understanding the pathophysiology of these syndromes.

Acute necrotizing encephalopathy (ANE) is a rare type of acute encephalopathy first described in Japan. The diagnosis is based on the topographic distribution and evolution of symmetric lesions visualized by computed tomography (CT) and magnetic resonance imaging (MRI) in the bilateral thalami and other specific brain areas [2]. Although the pathogenesis remained obscure, vasculopathy with breakdown of the blood–brain barrier (BBB) was suggested [3].

Recently, we established a novel method to identify cell-surface autoantigens, which we named Serological identification system for Autoantigens using a Retroviral vector and Flow cytometry (SARF) [4,5]. Using SARF, we successfully identified ephrin type B receptor 2 (EphB2), which has critical functions in neuronal and endothelial cells (ECs) [6], as a target of autoantibody from a patient with ANE complicated with systemic lupus erythematosus (SLE).

## Methods

### Source of human serum

Forty-eight patients with SLE (44 female and 4 male patients) diagnosed according to the 1997 revised criteria for the classification [7] were enrolled in this study. The patients gave written consent after the purpose and potential risks involved in the study were explained. The study protocol complied with the principles of the Declaration of Helsinki and was approved by the Ethics Committee of Tohoku University Graduate School of Medicine.

### Cell culture

Human umbilical vein ECs (HUVECs), Plat-E packaging cells, rat myeloma cells: YB2/0, and medium were purchased and grown as described previously [4]. Human brain microvascular ECs (HBMECs) and medium were purchased from Cell Systems (Kirkland, WA, USA).

### Flow cytometry

Binding activities of antibodies to the surface of ECs and EphB2 were measured and analyzed as described previously [4]. Briefly, aliquots of 1 × 10^5^ cells/tube were incubated in blocking buffer with primary antibodies at 4°C for 30 minutes. After washing, cells were incubated with secondary antibodies and 7-amino-actinomycin D (7-AAD) (BD Biosciences, Bedford, MA, USA) at 4°C for 30 minutes and analyzed by flow cytometry.

Goat anti-human EphB2 antibody was purchased from R&D Systems (Minneapolis, MN, USA), and recombinant EphB2 was purchased from Sino Biological (Beijing, China).

### SARF

Generation of HUVEC cDNA library and screening of cDNA were performed as described previously [4]. Briefly, the HUVEC cDNA library was retrovirally transfected into the YB2/0 cells. YB2/0 cells expressing the HUVEC cDNA library were incubated with serum IgG and fluorescein isothiocyanate (FITC)-labeled secondary antibody. Cells showing a high level of FITC fluorescence were sorted with FACSAriaII (Becton Dickinson, Franklin Lakes, NJ, USA). Sorted cells were kept in culture until the cell number increased sufficiently for the next round of sorting. Subcloning of cells was performed by the limiting dilution method. Total RNAs of cloned cells and unsorted cells were generated and subjected to microarray analysis (GeneChip Human Genome U133 Plus 2.0 Array; Affymetrix, Santa Clara, CA, USA).

### Expression of EphB2 in YB2/0 cells

The full-length EphB2 fragment was amplified by PCR from genomic DNA of EphB2-expressing YB2/0 clone sorted as described above, using Phusion High-Fidelity DNA Polymerase (Finnzymes, Keilaranta, Espoo, Finland). Primer sequences were as follows: 5′-AAGCGCAGCCATGGCTCT-3′, 3′-AGGCAGGTGAATGTCAAACC-5′. EphB2 fragment was inserted into the pMX-IRES-GFP vector (Cell Biolabs, San Diego, CA, USA).

## Case presentation

### Case of ANE complicated with SLE

**Figure 1:**
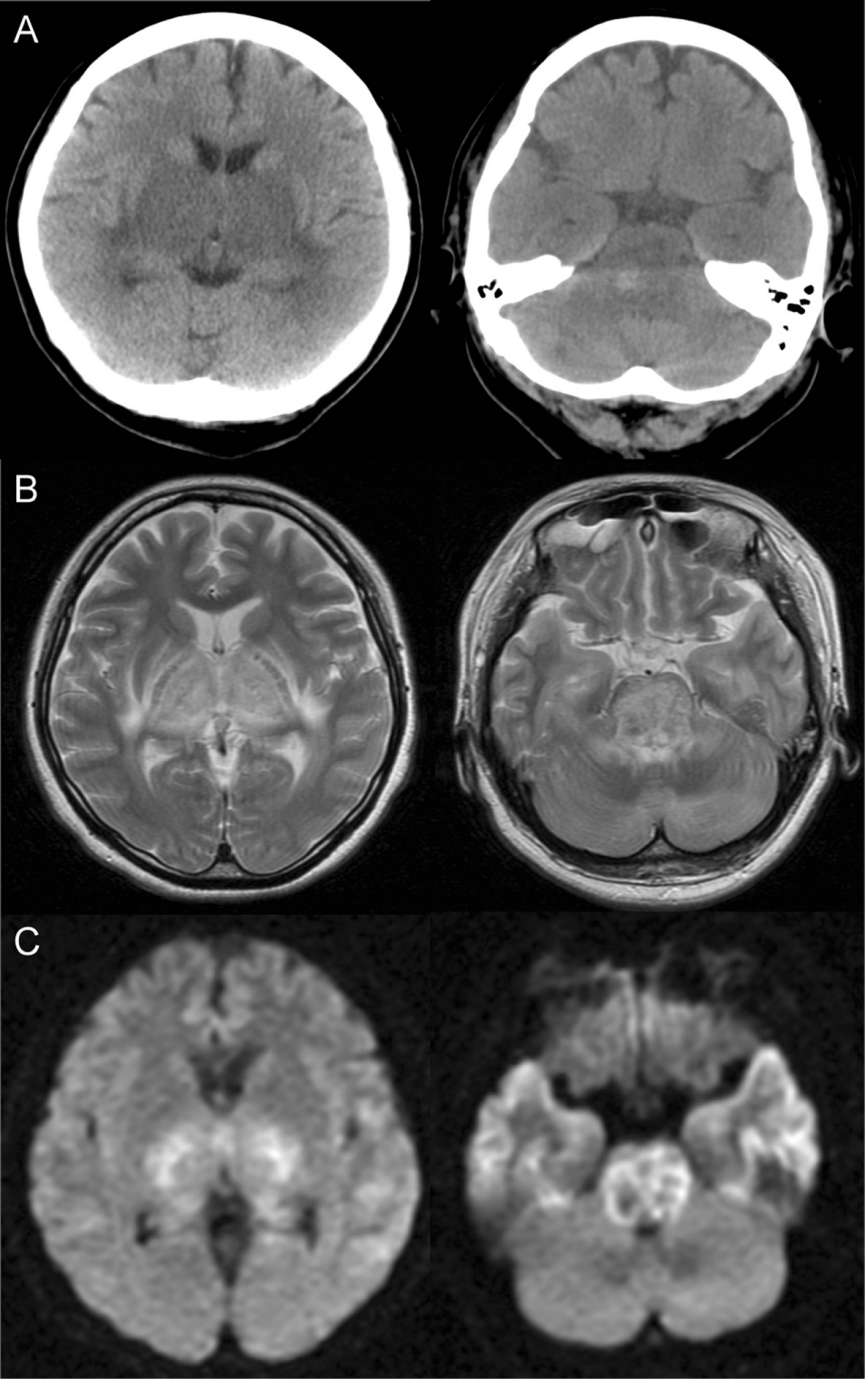
**Clinical imaging. (A)** CT showed low density area in the bilateral thalami, basal ganglia, medial temporal lobe, and brainstem. The brainstem was swollen, and high density area was observed in the pons. **(B)** MRI (T2-weighted image) revealed high signal intensity in the bilateral thalami, basal ganglia, medial temporal lobe, and brainstem. **(C)** MRI (diffusion-weighted image) also showed high signal intensity in the same area.

A 39-year-old Japanese woman was admitted to our hospital because of coma. One day previously, she had been admitted to another hospital because of diarrhea lasting three days. The next morning, she suddenly fell into a coma and developed quadriplegia, and was transferred to our hospital. On admission, her consciousness was GCSE1V1M2, both pupil sizes were 3 mm, and both pupillary light reflexes were absent. Decorticate rigidity and Babinski reflex were present. The cerebrospinal fluid (CSF) showed normal glucose and no pleocytosis, but the total protein level was elevated to 316 mg/dL. Laboratory tests indicated lymphopenia (410/μL), and elevated levels of aspartate aminotransferase (AST; 136 IU/L), lactate dehydrogenase (LDH; 740 IU/L), and C-reactive protein (CRP; 0.9 mg/dL). Serum complement levels were decreased as follows: C3, 42 mg/dL; C4, 5.3 mg/dL CH50, 10.8 U/mL. Antinuclear antibody (ANA) (×640, speckled) and anti-Sm antibody (48.7 index) were positive, but anti-dsDNA antibody and anti-phospholipid antibodies were negative. Tests for infections and urine were negative. CT showed low density area in the bilateral thalami, basal ganglia, medial temporal lobe, and brainstem (Figure 1A). MRI revealed high signal intensity in T2 (Figure 1B) and diffusion-weighted images in the same areas as described above (Figure 1C). A diagnosis of neuropsychiatric SLE (NPSLE) manifesting ANE was made based on the clinical, serological, and imaging findings. Despite extensive treatment, her consciousness did not recover, and she remained in a permanently vegetative state.

### Identification of EphB2 as a novel cell-surface autoantigen

**Figure 2:**
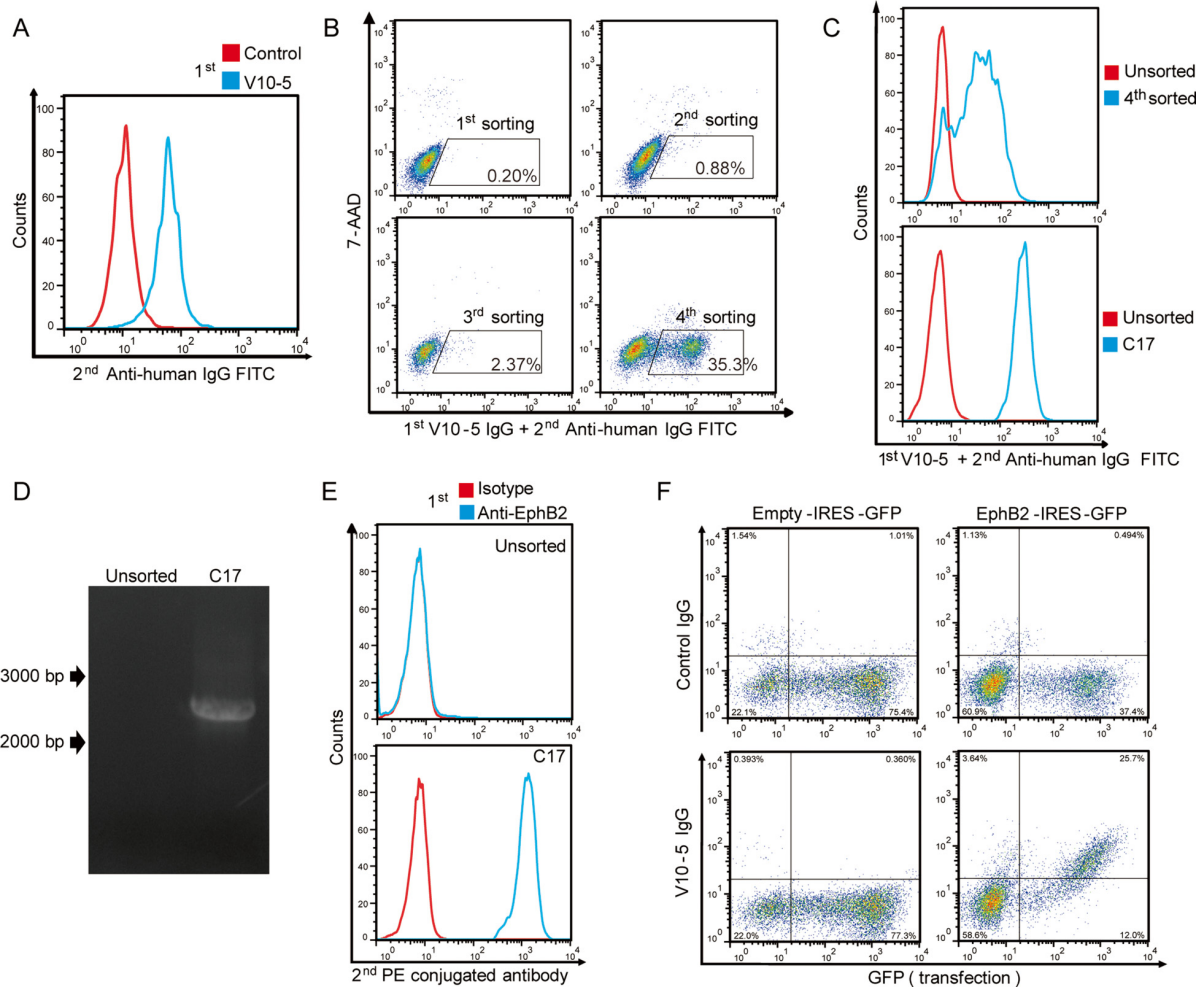
**Identification of ephrin type B receptor 2 (EphB2) as a novel autoantigen. (A)** Nonpermeabilized human umbilical vein endothelial cells (HUVECs) were stained with 0.5 mg/mL of IgG of control or V10-5 followed by secondary antibody and analyzed by flow cytometry. **(B)** HUVEC cDNA-expressing cells were stained with 0.5 mg/mL of V10-5 IgG followed by secondary antibody, and cells in the positive fraction were sorted (squares). **(C)** Unsorted cells and those at the fourth round of sorting (upper), and unsorted and cloned cells from those at the fourth round of sorting, C17 (lower), were stained with 0.5 mg/mL of V10-5 IgG followed by secondary antibody, and analyzed by flow cytometry. **(D)***EphB2* cDNA fragments inserted into the genomic DNA of C17 were amplified, and PCR products were electrophoresed on a 0.8 % agarose gel. **(E)** Unsorted and C17 were stained with isotype control or anti-EphB2 antibody, followed by secondary antibody and analyzed by flow cytometry. **(F)** Expression vector, empty-IRES-GFP, or EphB2-IRES-GFP was transfected into YB 2/0 cells, and the cells were stained with 0.5 mg/mL of control IgG or V10-5 IgG, followed by secondary antibody and analyzed by flow cytometry.

Serum from this patient (V10-5) showed strong binding activity to HUVECs (anti-endothelial cell antibody (AECA) activity) (Figure 2A). Using SARF, the YB2/0 cell line expressing HUVEC cDNA was incubated with V10-5 IgG and FITC-conjugated secondary antibody, and cells with strong FITC signals were sorted by flow cytometry (Figure 2B). After cell expansion, we repeated three more rounds of cell sorting. After the fourth round of sorting, cells bound to V10-5 IgG were markedly increased (Figure 2C, upper). Then, one clone (C17) was established from the V10-5 IgG-binding cell population by the limiting dilution method (Figure 2C. lower).

Microarray analysis of unsorted YB-2/0 and C17 indicated that the signal of EphB2 was significantly increased (2^6.16^-fold) in C17 compared to unsorted cells, and we confirmed that the *EphB2* cDNA was inserted into the genomic DNA of the V10-5-C17 clone by PCR (Figure 2D). We also confirmed the expression of EphB2 on the V10-5-C17 clone by flow cytometry (Figure 2E). Next, we generated an expression vector for EphB2, which was transfected into YB2/0 cells. V10-5 IgG showed significant binding activity to EphB2-expressing YB2/0 cells (Figure 2F), indicating that V10-5 IgG has anti-EphB2 activity. Thus, EphB2 was identified as a novel autoantigen in a patient with ANE complicated with SLE.

**Figure 3:**
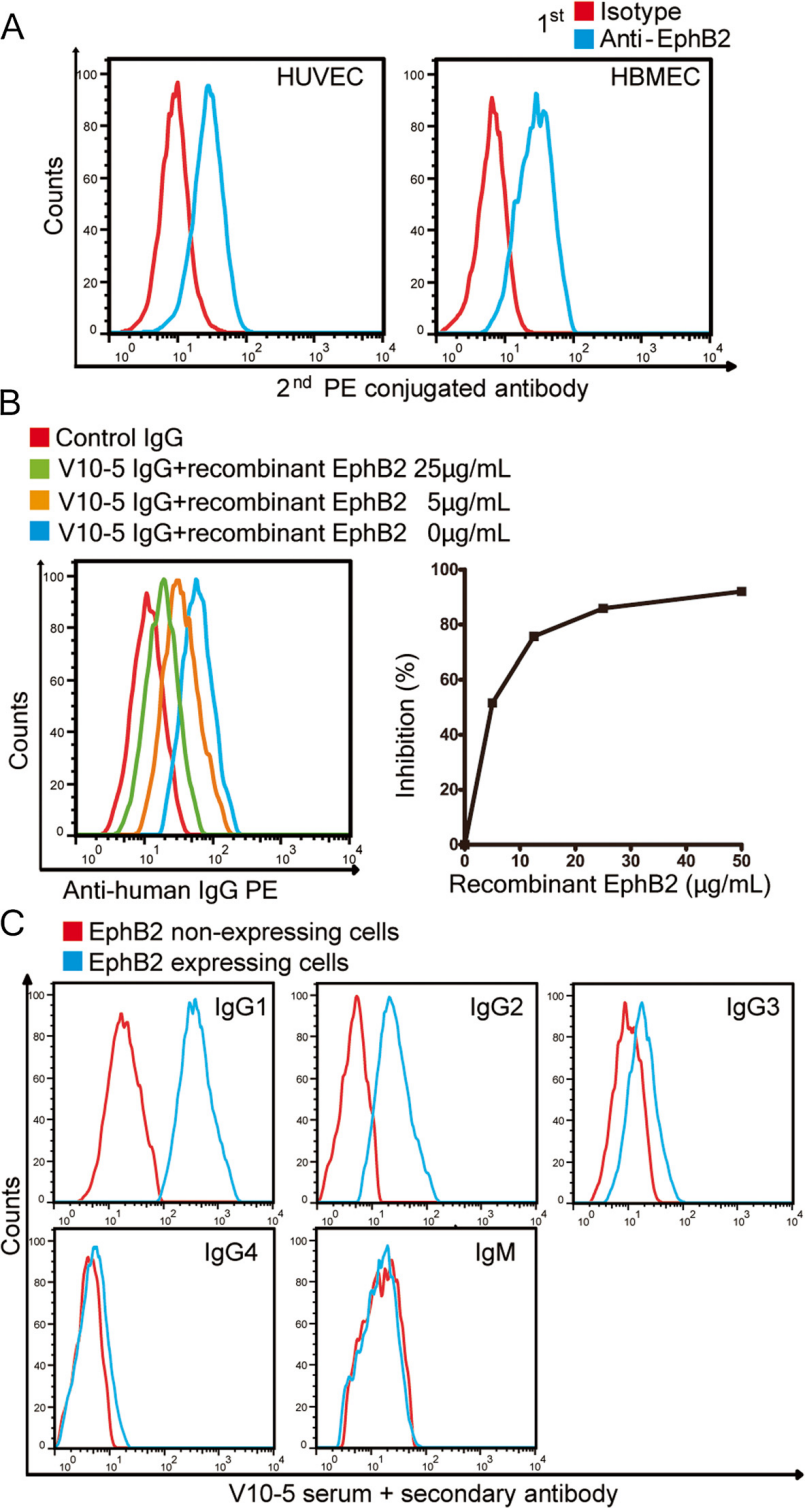
**Confirmation of anti-EphB2 activity of V10-5 IgG. (A)** Nonpermeabilized human umbilical vein endothelial cells (HUVECs) and human brain microvascular cells (HBMECs) were stained with isotype control or anti-EphB2 antibody followed by secondary antibody, and analyzed by flow cytometry. **(B)** Inhibition tests of binding activities to HBMECs were performed using V10-5 IgG with soluble EphB2 at the indicated concentrations. Control indicates binding activity of IgG from healthy donor to HBMECs. **(C)** EphB2-expressing or non-expressing YB2/0 cells were stained with 1:10 diluted V10-5 serum followed by secondary antibody against human IgG1, IgG2, IgG3, IgG4, or IgM, and analyzed by flow cytometry.

Flow cytometry indicated that HUVECs and HBMECs also expressed EphB2 on their cell surfaces (Figure 3A). We conducted inhibition tests to determine whether the AECA activity of V10-5 IgG was due to anti-EphB2 activity. Anti-HBMEC activity of V10-5 IgG was inhibited by addition of recombinant EphB2 (Figure 3B). We also analyzed the IgG subclasses of anti-EphB2 antibody by flow cytometry. IgGs with anti-EphB2 activity were IgG1, IgG2, and IgG3 (Figure 3C).

Although we measured anti-EphB2 activity among 47 SLE patients (nine patients had been diagnosed with NPSLE, none of them presented ANE) who had AECA activities, anti-EphB2 antibody was not detected in any other SLE patients examined.

## Discussion

Identification of EphB2 as a novel autoantigen may be important in clarifying the pathogenesis of ANE because anti-EphB2 antibody has potential to play pathogenic roles in neuronal and vascular lesions.

ANE has a high mortality rate and is usually preceded by infections, such as influenza, with the signs of brain dysfunction appearing a few days later [8]. It is characterized by symmetrical brain necrosis, which involves the thalamus followed by the tegmentum of the pons and other regions, and these are demonstrated by CT and MRI [3]. Neuropathologically, the lesions tend to spread along small blood vessels, and the extent of the lesions appears to be determined by the spread of edema fluid. These observations indicate the vasogenic nature of edema-necrosis in ANE, resulting from local breakdown of the BBB [8]. EC necrosis and inflammatory fibrinoid vasculitis have also been reported [9].

Although our patient showed characteristic features of ANE, this is a very rare complication of SLE; to our knowledge, no other cases of SLE manifesting ANE have been reported to date. Although some previous reports indicated the absence of ANA in ANE [2], the correlation between SLE and ANE is uncertain.

Eph receptors belong to one of the largest families of tyrosine kinases. They are known to have diverse functions in neural development, cell morphogenesis, tissue patterning, angiogenesis, and neural plasticity [6]. Among them, the critical roles of EphB2 in the nervous system have recently been clarified. The important functions of EphB2 include regulation of NMDAR-dependent Ca^2+^ influx and downstream transcription factors in neuronal cells [10], and the involvement in neurological diseases, including Alzheimer’s disease, anxiety, and anti-NMDA encephalitis has been reported [11-13]. We performed an immunohistochemical study of mouse brain tissues using a commercially available anti-EphB2 antibody. As a result, we identified positive reactions on neural cells and glia in the cerebral cortex and also ECs in the subarachnoid regions (data not shown). Further studies would be done to investigate whether the reactivity of the patient’s antibodies with brain is abrogated by immunoadsorption with EphB2 or in EphB2 knockout mice tissues. Interestingly, EphB2 is also expressed on ECs and is required for EC formation of cord-like structures [14].

Thus, anti-EphB2 antibody could potentially interfere not only with ECs including those of the BBB (acting as an AECA), but also neuronal cells (acting as an anti-neuronal antibody) if the BBB has been breached. Taken together, the possible suspected pathomechanisms are 1) anti-EphB2 antibody damages vascular ECs, which results in breakdown and increased permeability of BBB; 2) anti-EphB2 antibody exudated into brain tissue binds neurons and glia, which causes neuronal dysfunction and brain necrosis.

In the present study, anti-EphB2 antibody was not detected in any other patients with SLE, none of whom manifested ANE. Although we did not evaluate the prevalence of anti-EphB2 antibody in other ANE patients because of its rarity, anti-EphB2 antibody may define a novel group of brain disorders the clinical manifestations of which are similar to those of ANE. If this is the case, anti-EphB2 antibody may be a useful biomarker, and provide new insight into such brain disorders.

## Conclusion

We identified EphB2 as a novel autoantigen in a patient with ANE complicated with SLE. Anti-EphB2 antibody may have dual activity as an AECA and an anti-neuronal antibody. Further studies are needed to determine the clinical prevalence and specificity of anti-EphB2 antibody, and the molecular mechanisms by which anti-EphB2 antibody mediates neuronal and vascular pathological lesions.

## Consent

Written informed consent was obtained from the next of kin of this patient for publication of this case report and any accompanying images because consciousness of this patient did not recover. A copy of the written consent is available for review by the Editor-in-Chief of this journal.

## Abbreviations

AECA: Anti-endothelial cell antibody; AMPAR: α-amino-3-hydroxy-5-methyl-4-isoxazolepropionic acid receptor; ANA: Antinuclear antibody; ANE: Acute necrotizing encephalopathy; AST: Aspartate aminotransferase; BBB: Blood–brain barrier; Caspr2: Contactin-associated protein-like 2; CRP: C-reactive protein; CSF: Cerebrospinal fluid; CT: computed tomography; ECs: Endothelial cells; EphB2: Ephrin type B receptor 2; FITC: Fluorescein isothiocyanate; GABAB: γ-aminobutyric acid receptor-B; GlyR: Glycine receptor; HBMECs: Human brain microvascular endothelial cells; HUVECs: Human umbilical vein endothelial cells; LDH: Lactate dehydrogenase; LGI1: Leucine-rich glioma-inactivated protein 1; mGluR: Metabotropic glutamate receptor; MRI: Magnetic resonance imaging; NMDAR: N-methyl-D-aspartate receptor; NPSLE: Neuropsychiatric systemic lupus erythematosus; PCR: Polymerase chain reaction; SARF: Serological identification system for Autoantigens using a Retroviral vector and Flow cytometry; SLE: Systemic lupus erythematosus; 7-AAD: 7-amino-actinomycin D.

## Competing interests

The authors declare that they have no competing interests.

## Authors’ contributions

TS carried out the molecular biological studies, flow cytometry, clinical evaluation, and drafted the manuscript. HF, MO, and MN participated in design of the study, performed the molecular biological studies, and helped to draft the manuscript. RW, YS, SS, and TI participated in the design of the study and helped to draft the manuscript. HH conceived the study, participated in its design and coordination, and helped to draft the manuscript. All authors have read and approved the final manuscript.
